# Highly specific and ultrasensitive plasma test detects Abeta(1–42) and Abeta(1–40) in Alzheimer’s disease

**DOI:** 10.1038/s41598-021-89004-x

**Published:** 2021-05-06

**Authors:** Elisabeth H. Thijssen, Inge M. W. Verberk, Jeroen Vanbrabant, Anne Koelewijn, Hans Heijst, Philip Scheltens, Wiesje van der Flier, Hugo Vanderstichele, Erik Stoops, Charlotte E. Teunissen

**Affiliations:** 1 Neurochemistry Laboratory, Department of Clinical Chemistry, Amsterdam Neuroscience, Vrije Universiteit Amsterdam, Amsterdam UMC, Amsterdam, The Netherlands; 2 ADx NeuroSciences, Ghent, Belgium; 3 Alzheimer Center Amsterdam, Department of Neurology, Amsterdam Neuroscience, Vrije Universiteit Amsterdam, Amsterdam UMC, Amsterdam, The Netherlands; 4 Biomarkable, Gent, Belgium

**Keywords:** Immunochemistry, Proteins, Blood proteins, Diseases of the nervous system, Molecular neuroscience, Biomarkers, Diagnostics, Target identification, Diagnostic markers, Neurological disorders, Biochemistry, Drug discovery, Neuroscience, Biomarkers, Neurology

## Abstract

Plasma biomarkers that reflect specific amyloid beta (Abeta) proteoforms provide an insight in the treatment effects of Alzheimer’s disease (AD) therapies. Our aim was to develop and validate ready-to-use Simoa ‘Amyblood’ assays that measure full length Abeta_1-42_ and Abeta_1-40_ and compare their performance with two commercial assays. Linearity, intra- and inter-assay %CV were compared between Amyblood, Quanterix Simoa triplex, and Euroimmun ELISA. Sensitivity and selectivity were assessed for Amyblood and the Quanterix triplex. Clinical performance was assessed in CSF biomarker confirmed AD (n = 43, 68 ± 6 years) and controls (n = 42, 62 ± 5 years). Prototype and Amyblood showed similar calibrator curves and differentiation (20 AD vs 20 controls, *p* < 0.001). Amyblood, Quanterix triplex, and ELISA showed similar linearity (96%-122%) and intra-assay %CVs (≤ 3.1%). A minor non-specific signal was measured with Amyblood of + 2.4 pg/mL Abeta_1-42_ when incubated with 60 pg/mL Abeta_1-40_. A substantial non-specific signal of + 24.7 pg/mL Abeta_x-42_ was obtained when 40 pg/mL Abeta_3-42_ was measured with the Quanterix triplex. Selectivity for Abeta_1-42_ at physiological Abeta_1-42_ and Abeta_1-40_ concentrations was 125% for Amyblood and 163% for Quanterix. Amyblood and Quanterix ratios (*p* < 0.001) and ELISA Abeta_1-42_ concentration (*p* = 0.025) could differentiate AD from controls. We successfully developed and upscaled a prototype to the Amyblood assays with similar technical and clinical performance as the Quanterix triplex and ELISA, but better specificity and selectivity than the Quanterix triplex assay. These results suggest leverage of this specific assay for monitoring treatment response in trials.

## Introduction

Alzheimer’s disease (AD) is the most common form of dementia, affecting 50 million people worldwide^1^ In vivo AD diagnosis and monitoring of treatment response in clinical trials is based on changes in amyloid beta proteins (Abeta), measured by positron emission tomography (PET) or in cerebrospinal fluid (CSF)^2–4^ However, both methods are associated with major disadvantages in terms of high costs (PET) and invasiveness (lumbar puncture to collect CSF), which prohibit repetitive analysis. Therefore, it is critical to have access to blood-based biomarkers as a less invasive method for monitoring of treatment effects.

Since the introduction of highly sensitive technologies such as bead-based immunoassays or immunoprecipitation combined with mass spectrometry (IP-MS), consistent reductions in plasma Abeta_42/40_ ratio are reported in AD^5–10^ With IP-MS, a 10–15% reduction in the plasma Abeta_42/40_ ratio has been measured in amyloid PET or CSF positive cases, compared to amyloid negative cases^7^, and the ratio could differentiate the groups with an area under the curve (AUC) of 84–97%^8^ IP-MS is often more sensitive than immunoassays, but requires a high sample volume and a high level of expertise which may limit broad clinical implementation. The Abeta_42/40_ ratio measured with the Single Molecule Array (Simoa, Quanterix)^11^, showed promising results in identifying individuals with amyloid pathology (AUC of 68–87%^5,9^) across controls, mild cognitive impairment (MCI), and AD. This bead-based technology offers high throughput and is ultrasensitive. However, the commercial Quanterix assay lacks antibody specificity, since it measures Abeta_x-42_ and Abeta_x-40_, whereas the specificity for full-length Abeta_1-42_ and Abeta_1-40_ is important to understand the effect of treatments that target specific Abeta isoforms^12^.

At Amsterdam University Medical Centers (Amsterdam UMC) and ADx Neurosciences (ADx) novel ready to use (RTU) immunoassays, called “Amyblood”, were developed for detection of full length Abeta_1-42_ and Abeta_1-40_ with Simoa technology^13^ This way both the high specificity of the antibodies and the high sensitivity of Simoa technology could be leveraged. The aim of the present study is to analytically validate the Amyblood assays, including specificity and selectivity evaluation, and explore their clinical value in a CSF-biomarker confirmed cohort of AD patients and controls. We compared the analytical and clinical performance of the Amyblood assays with the commercially available Quanterix triplex kit (Abeta_x-42_/Abeta_x-40_) and the Euroimmun ELISA assays (Abeta_1-42_/Abeta_1-40_).

## Results

### Robustness of the upscaled Amyblood RTU assays

Differences in antibody lot were determined by comparing three lots of ADx102 and ADx103 conjugated bead batches. The variation in AEB values of the calibrator across the lots was 19%CV for Abeta_1-42_ and 14% for Abeta_1-40_ (eFigure 1). The calibration curves %CV of the small and upscaled bead batches based on calibrator AEB values was 10.0% (range: 3.8%-17.6%) for Abeta_1-42_ (eFigure 2A) and 7.0% (range: 0.03%-13.9%) for Abeta_1-40_ beads (eFigure 2B). Amyblood Abeta_1-42/1–40_ inter-assay %CV was 2.9% (sample 1: 3.9% at AUMC, 3.5% ADx, sample 2: 2.2% at AUMC, 2.0% at ADx) and inter-center %CV was 17.2% (sample 1: 14% at AUMC, 23% ADx, sample 2: 14% at AUMC, 13% at ADx) (eFigure 3).

**Figure 1:**
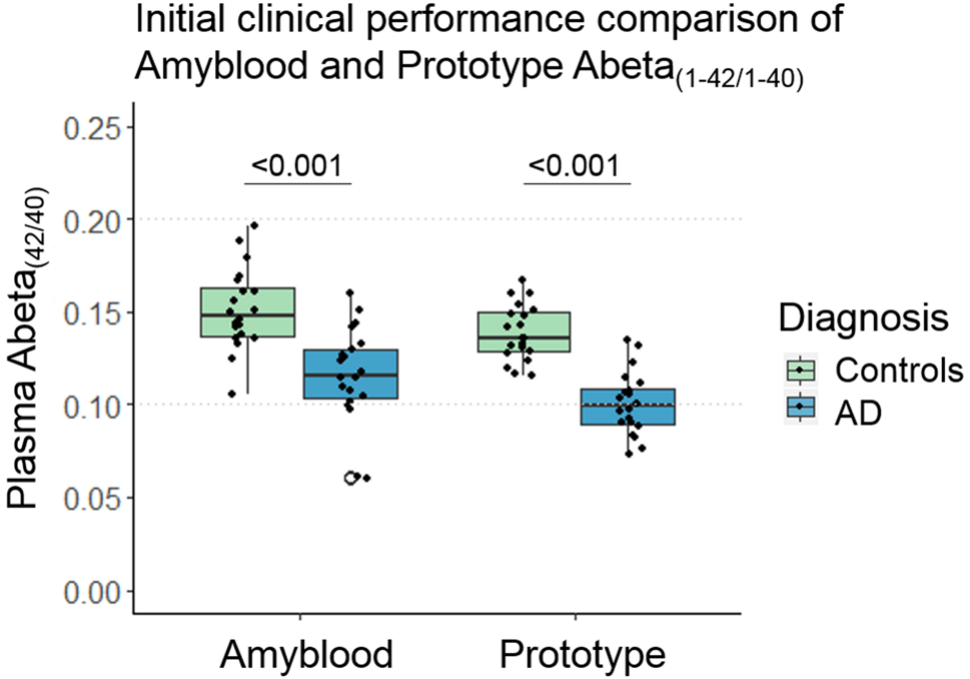
Abeta_1-42/1–40_ ratio measured in AD and control samples with the Amyblood and Prototype assays. Comparison of the prototype and Amyblood assays was based on measuring a feasibility sample set of 40 samples, 20 CSF Abeta_1-42_ positive AD cases and 20 CSF Abeta_1-42_ negative controls, measured in duplicate in both assays. The average Abeta_1-42/1–40_ ratio measured with the Amyblood assays were 0.151 ± 0.022 for controls and 0.116 ± 0.026 for AD, the average Abeta_1-42/40_ ratio measured with the prototype was 0.139 ± 0.015 for controls and 0.100 ± 0.017 for AD.

For proof of concept, the Abeta_1-42_ and Abeta_1-40_ concentrations were measured in a sample set of 20 CSF Abeta_1-42_ positive AD cases and 20 CSF Abeta_1-42_ negative SCD cases in both these upscaled versions and the prototype assays that were basis for these upscaled assays. This prototype assay (supplementary methods) developed in-house by Amsterdam UMC similarly utilized the capture antibodies ADx102^14^ for Abeta_1-42_ or ADx103^14^ for Abeta_1-40_ and detector antibody ADx101^14^ for Abeta_1_ (ADx, Ghent, BE). The fold change in Abeta_1-42/1–40_ ratio between AD and controls was 1.39 for the prototype and 1.29 for the Amyblood (both: *p* < 0.001, Fig. 1). There was a good correlation between the prototype and Amyblood results of Abeta_1-42_ (R = 0.77, *p* < 0.001), Abeta_1-40_ (R = 0.89, *p* < 0.001), and the Abeta_1-42/1–40_ ratio (R = 0.69, *p* = 0.001) (eFigure 4).

### Analytical validation of all six assays in this study

Upon serial dilution, all six Abeta assays showed acceptable linearity (i.e. in the range 80–120%), except for a small deviation of the Quanterix Abeta_x-42_ assay (122%) (eTable 1). All assays showed intra-assay %CV values < 10% for clinical samples. The inter-assay %CV of the Amyblood Abeta_1-42_ assay was 13.3%, and 10.4% for Abeta_1-40_. The inter-assay %CV of Quanterix Abeta_x-42_ assay was 7.9%, and 5.4% for Abeta_x-40._ The LLOQ’s were 1.6 pg/mL for Amyblood Abeta_1-42_ and 1.7 pg/mL for Abeta_1-40_; 0.34 pg/mL for Quanterix Abeta_x-42_ and 0.16 pg/mL for Abeta_x-40_; and 5.4 pg/mL for ELISA Abeta_1-42_ and 11.9 pg/mL for Abeta_1-40_. One sample measured with the Amyblood Abeta_1-42_ assay and eight samples measured with the ELISA Abeta_1-42_ assay were lower than their blank. All Abeta_40_ concentrations were above the blank (eFigure 5).

### Assay specificity and selectivity

Selectivity and specificity of the Abeta_42_ and Abeta_40_ measurements were tested for the Amyblood and Quanterix assays. Detailed specificity concentrations and %recovery are described in eTables 2 and 3. Different spike concentrations were used for the Amyblood and Quanterix assays, to align with their difference in absolute Abeta_42_ and Abeta_40_ concentrations (eTable 4).

### Specificity of the Amyblood and Quanterix assays

**Figure 2:**
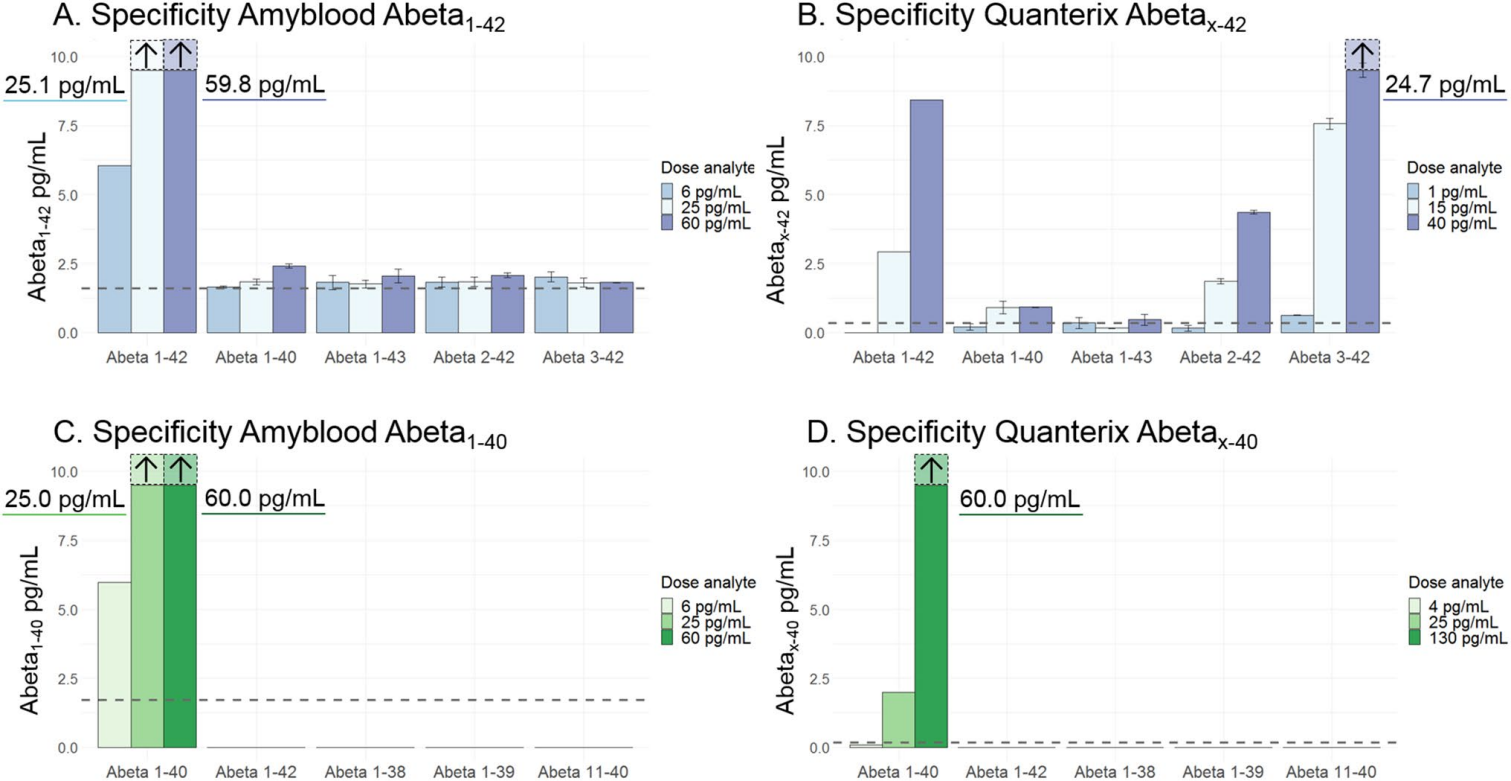
Specificity of the Amyblood and Quanterix assays. (**A**) Amyblood Abeta_1-42_ concentrations were measured above blank reading for all abeta fragments, with a maximum of 2.4 pg/mL for 60 pg/mL of Abeta_1-40_ for the fragments other than Abeta_1-42_. (**B**) Quanterix Abeta_x-42_ signal were measured above blank for Abeta_2-42,_ and Abeta_3-42_, with a maximum of 24.7 pg/mL for 40 pg/mL of Abeta_3-42_ (**C**) No increase in Amyblood Abeta_1-40_ signal was measured above blank reading for any of the Abeta fragments other than Abeta_1-40_. (**D**) No increase in Quanterix Abeta_x-40_ concentration was measured above blank reading for any of the Abeta fragments other than Abeta_1-40_. Dashed line represents the LLOQ per assay.

Low, medium and high concentrations of Abeta fragments 1–42, 1–40, 1–43, 2–42, and 3–42 spiked in sample buffer were measured with the Abeta_1-42_ and Abeta_x-42_ assays. For Amyblood, a minor signal above blank was measured when other Abeta fragments were incubated, with a maximum reported result of 2.4 pg/mL for 60 pg/mL of spiked Abeta_1-40_ (Fig. 2A). A substantial increase compared to the blank was observed for Quanterix Abeta_x-42_ values, especially for Abeta_2-42,_ and Abeta_3-42_ fragments, with a maximum of + 24.7 pg/mL Abeta_x-42_ for 40 pg/mL of Abeta_3-42_ (Fig. 2B). When measuring Abeta fragments 1–42, 1–38, 1–39, and 11–40 with the Amyblood Abeta_1-40_ and Quanterix Abeta_x-40_ assays, none of the other Abeta isoforms yielded a signal above blank. The Abeta 1–40 fragment was accurately measured with the Amyblood assay, but yielded a lower formal concentration when measured with the Quanterix assay (Fig. 2C and D).

### Selectivity of the Amyblood and Quanterix assays

We tested selectivity by measuring the signal of one analyte in sample diluent (either Abeta_1-42_ or Abeta_1-40_), in the presence of a known concentration of the other analyte. Described physiological concentrations were based on concentrations measurements in plasma after sample dilution (eTable 4).

**Figure 3:**
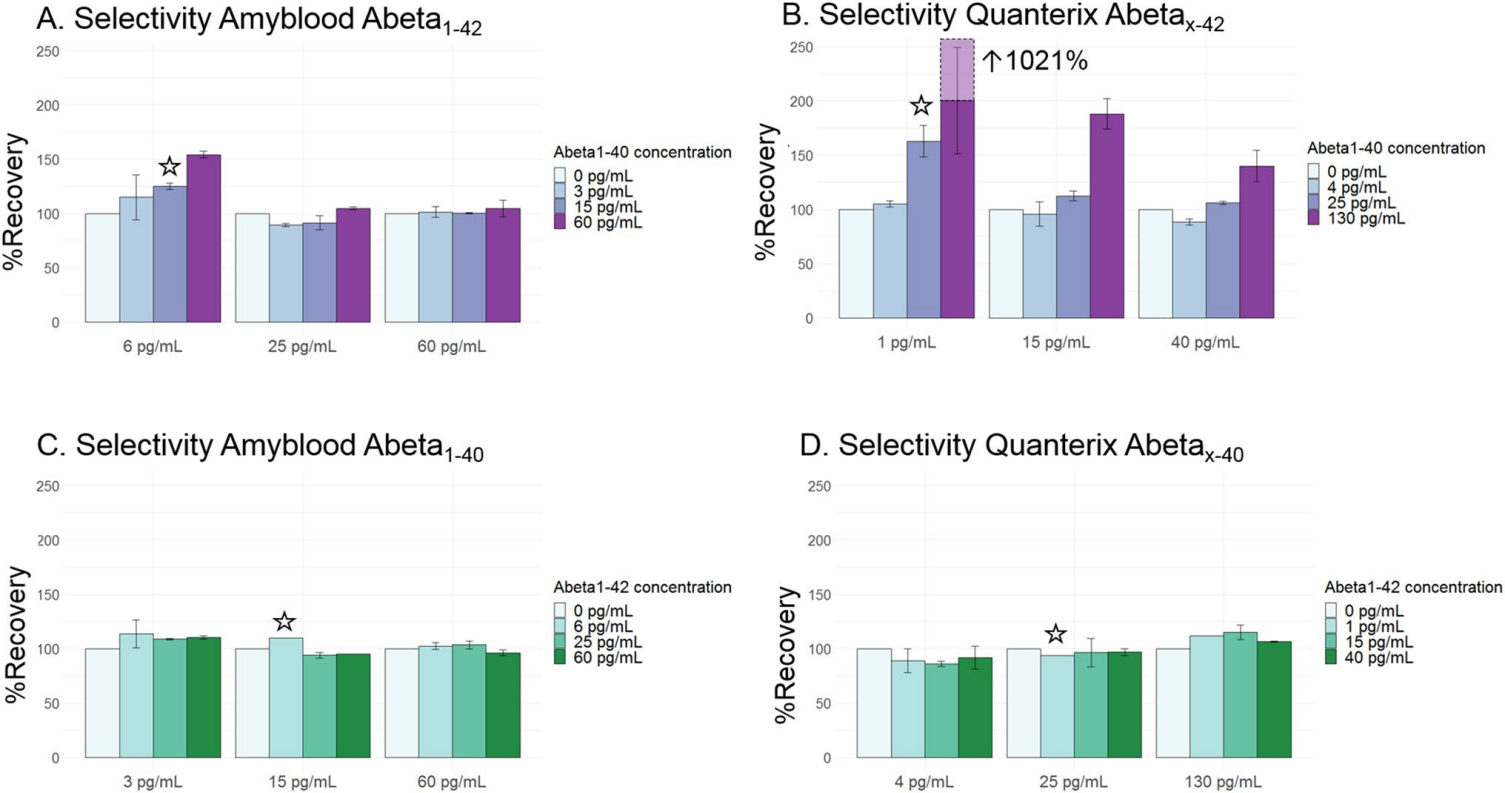
Selectivity of the Amyblood and Quanterix amyloid assays. (**A**) The highest %recovery measured with the Amyblood Abeta_1-42_ assay was 125% in the presence of 6 pg/mL Abeta_1-42_ and 15 pg/mL Abeta_1-40_. (**B**) The highest %recovery measured with the Quanterix Abeta_x-42_ assay was 1021% in the presence of 1 pg/mL Abeta_1-42_ and 130 pg/mL Abeta_1-40_. (**C**) The highest %recovery measured with the Amyblood Abeta_1-40_ assay was 110% in the presence of 3 pg/mL Abeta_1-40_ and 6 pg/mL Abeta_1-42_. (**D**) The highest %recovery measured with the Quanterix Abeta_x-40_ assay was 115% in the presence of 130 pg/mL Abeta_1-40_ and 15 pg/mL Abeta_1-40_. The concentrations in buffer nearest to physiological Abeta_1-42_ and Abeta_1-40_ combinations in plasma as measured with this assay are indicated with an asterisk.

For the Amyblood assay, the %recovery of 6 pg/mL Abeta_1-42_ measured with the Amyblood assay increased from 115% in the presence of the low concentration (3 pg/mL) of Abeta_1-40_ to 154% for the high concentration (60 pg/mL) of Abeta_1-40._ The %recovery of 25 pg/mL Abeta_1-42_ was 89% in presence of the low and 105% for in the presence of the high concentration Abeta_1-40._ The %recovery of 60 pg/mL Abeta_1-42_ was 101% in the presence of the low and 105% in the presence of the high Abeta_1-40_ concentration_._ At 6 pg/mL Abeta_1-42_ and 15 pg/mL Abeta_1-40_, which approximate the physiological concentrations measured in plasma after correction for dilution factor, the %recovery of Abeta_1-42_ was 125% (Fig. 3A).

For the Quanterix assay, the %recovery of 1 pg/mL plasma Abeta_x-42_ measured with the Quanterix assay increased from 105% in the presence of the low concentration (4 pg/mL) Abeta_1-40_ to 1021% in the presence of the high concentration (130 pg/mL) Abeta_1-40._ The %recovery of 15 pg/mL Abeta_1-42_ was 96% in the presence of a low and 188% in the presence of a high concentration of Abeta_1-40._ The %recovery of 40 pg/mL Abeta_1-42_ was 88% in the presence of the low concentration of Abeta_1-40_ and 140% in the presence of the high Abeta_1-40_ concentration_._ At 1 pg/mL Abeta_1-42_ and 25 pg/mL Abeta_1-40_, which approximate the physiological concentrations, the %recovery of Abeta_1-42_ was 163% (Fig. 3B). For both Abeta_42_ assays, the increase in %recovery returned to normal upon increasing Abeta_42_ concentrations relative to the Abeta_40_ concentration.

The %recovery of 3 pg/mL plasma Abeta_1-40_ measured with the Amyblood assay was 113% for the low concentration (6 pg/mL) Abeta_1-42_ and 110% for the high concentration (60 pg/mL) Abeta_1-42._ The %recovery of 15 pg/mL Abeta_1-40_ was 110% for the low and 95% for the high concentration Abeta_1-42._ The %recovery of 60 pg/mL Abeta_1-40_ was 102% for the low and 96% for the high Abeta_1-42._ At physiological concentrations of 15 pg/mL Abeta_1-40_ and 6 pg/mL Abeta_1-42_ the %recovery of Abeta_1-40_ was 110% (Fig. 3C).

The %recovery of 4 pg/mL plasma Abeta_x-40_ measured with the Quanterix assay was 89% for the low concentration (1 pg/mL) Abeta_1-42_ and 92% in the presence of the high concentration (40 pg/mL) Abeta_1-42._ The %recovery of 25 pg/mL Abeta_1-40_ was 94% in the presence of the low and 97% in presence of the high concentration Abeta_1-42._ The %recovery of 130 pg/mL Abeta_1-40_ was 112% for the low and 106% for the high concentration Abeta_1-42._ The highest %recovery was 115%, found at 130 pg/mL Abeta_1-40_ and 15 pg/mL Abeta_1-42_. At physiological concentrations of 25 pg/mL Abeta_1-40_ and 1 pg/mL Abeta_1-42_ the %recovery of Abeta_x-40_ was 94% (Fig. 3D). The %recovery of both Abeta_40_ assays seems relatively unaffected by an increase in Abeta_42_.

### Assay validation in clinical samples

**Figure 4:**
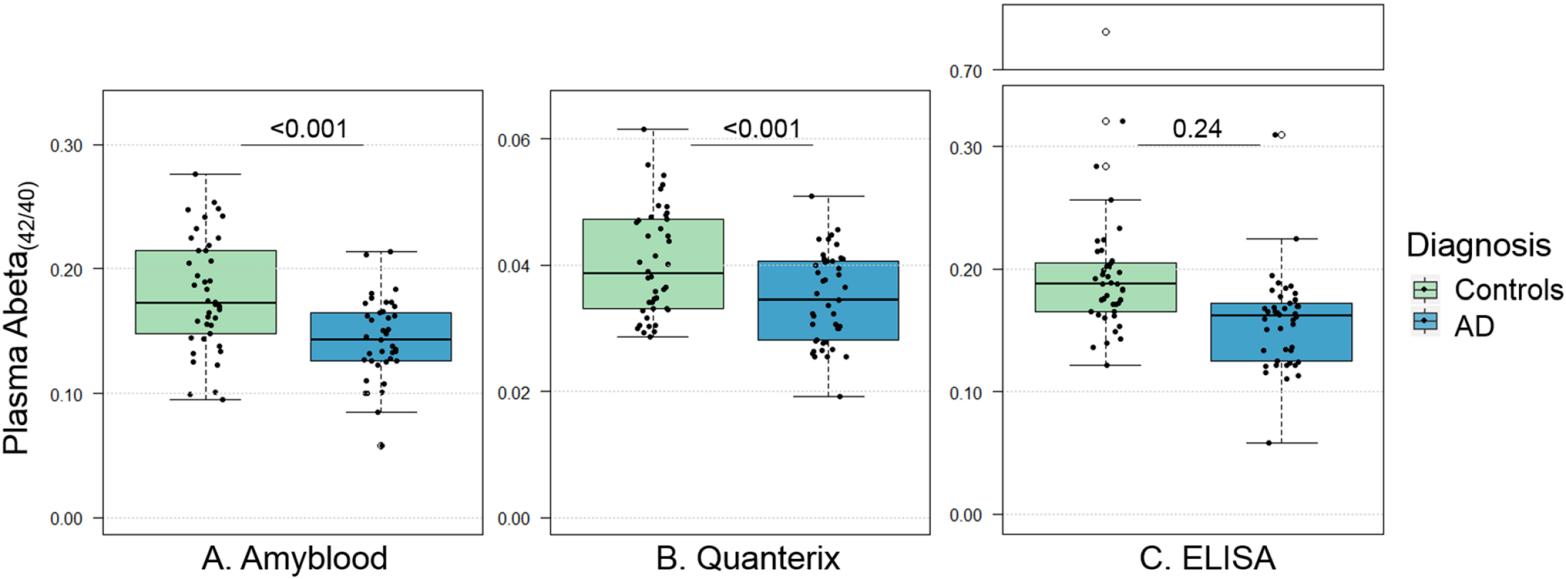
The plasma Abeta_42/40_ ratio measured with three different immunoassays. Plasma Abeta_42/40_ ratio’s measured in CSF Abeta_1-42_ negative controls and CSF Abeta_1-42_ positive AD groups. (**A**) Abeta_1-42/1–40_ measured with the Amyblood assays. (**B**) Abeta_x-42/x-40_ measured with the Quanterix triplex. (**C**) Abeta_1-42/1–40_ measured with the Euro-immun ELISA assays. P-values are corrected for sample batch, sample storage time, age, and sex.

Plasma Abeta_42_ was reduced in AD patients compared to controls for all three assays: -8.5% for Amyblood (uncorrected: *p* = 0.075; corrected for age, sex, storage time, and sample run: *p* = 0.012), -3.6% for Quanterix (uncorrected *p* = 0.096, corrected *p* = 0.005), and -9.5% for ELISA (uncorrected *p* = 0.008, corrected *p* = 0.025). There were no differences in plasma Abeta_40_ levels between AD patients and controls after correction for the indicated covariates: Amyblood; uncorrected: *p* = 0.023; corrected: *p* = 0.98, Quanterix: uncorrected: *p* = 0.16, corrected: *p* = 0.96, ELISA: uncorrected: 0.06, corrected: 0.62 (eTable 4, eFigure 6). The Amyblood Abeta_1-42/1–40_ ratio was decreased by -21% in AD patients compared to controls (*p* < 0.001 uncorrected, corrected *p* < 0.001). The Quanterix Abeta_x-42/x-40_ ratio was decreased by -13% (uncorrected *p* = 0.001, corrected *p* < 0.001). The ELISA Abeta_1-42/1–40_ ratio was decreased by -22% (uncorrected *p* = 0.004, corrected *p* = 0.24). Excluding a Abeta_1-42/1–40_ ELISA outlier that was 3.9 times the median ratio for controls from the statistical analysis changed the p-values for the ELISA analyses (uncorrected *p* < 0.001, corrected *p* = 0.06) (Fig. 4).

### Correlations of results of the three immuno-assays

**Figure 5:**
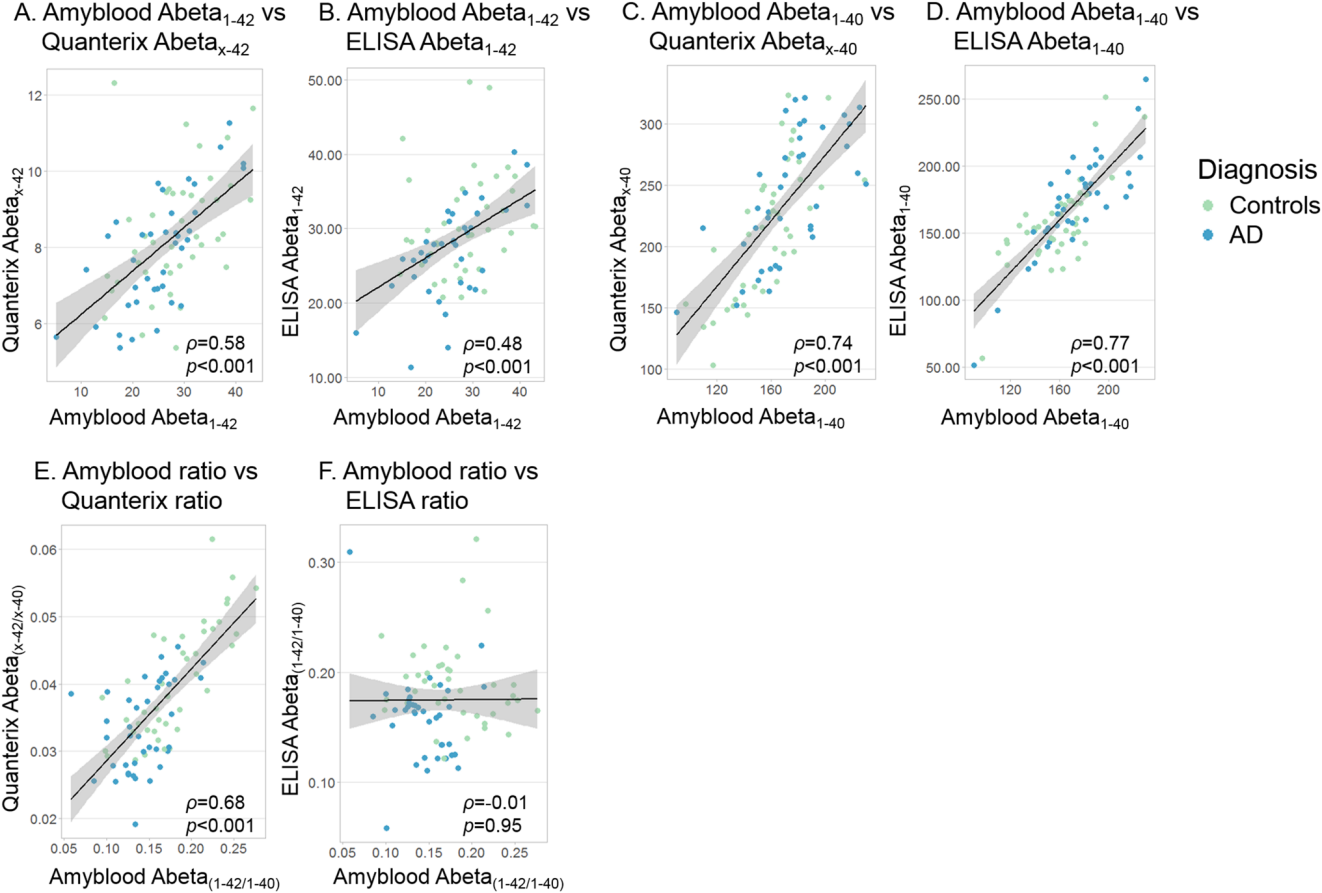
Correlations between Abeta_42,_ Abeta_40,_ and the ratio measured with Amyblood, Quanterix and ELISA. (**A, B**) Abeta_42_ concentrations measured with Amyblood were correlated with Quanterix and ELISA. (**C, D**) Abeta_40_ concentrations measured with Amyblood were correlated with Quanterix and ELISA. (**E, F**) The plasma Abeta_42/40_ ratio measured with the Amyblood and Quanterix assays correlated. The plasma Abeta_42/40_ ratio measured with ELISA did not correlate with Amyblood. The Abeta_42/40_ outlier in ELISA has been excluded from the figure for visualization, but was included in the calculated correlation coefficient. All correlations coefficients are Spearman’s rho.

The plasma Amyblood Abeta_42_ concentrations correlated with the Quanterix and ELISA Abeta_42_ results (*ρ* > 0.48, *p* < 0.001) (Fig. 5A and B). Amyblood Abeta_40_ concentrations correlated with the Quanterix and ELISA Abeta_40_ results (*ρ* > 0.74, *p* < 0.001) (Fig. 5C and D). The Amyblood Abeta_42/40_ ratio correlated with the Quanterix ratio (*ρ* = 0.68, *p* < 0.001), not with the ELISA ratio (*ρ* = -0.01, *p* = 0.95) (Fig. 5E and F).

The CSF Abeta_1-42_ concentrations did not correlate with plasma Abeta_42_ measured with Amyblood (*ρ* = 0.10, *p* = 0.36), Quanterix (*ρ* = 0.11, *p* = 0.32) or ELISA (*ρ* = 0.25, *p* = 0.09), whereas the CSF Abeta_1-42_ concentrations did correlate with the Abeta_42/40_ ratio, measured with Amyblood (*ρ* = 0.25, *p* = 0.02), Quanterix (*ρ* = 0.28, *p* = 0.01) and ELISA (*ρ* = 0.36, *p* = 0.001).

## Discussion

With this study we introduce the novel Amyblood assays developed on ultrasensitive Simoa technology for detection of the specific N-terminal Abeta peptides Abeta_1-42_ and Abeta_1-40_ in plasma. The validation was successful and the results were highly comparable to technical and clinical validation results obtained for two commercially available assays: the Quanterix triplex and Euroimmun ELISA. The specificity of the Amyblood assays for the specific isoforms was higher compared to the Quanterix assay. Moreover, the Amyblood Abeta_1-42/1–40_ ratio could successfully differentiate AD cases from controls, similar to the Quanterix assay. The ELISA ratio could not differentiate the groups, though the ELISA Abeta_1-42_ concentration by itself could. Our validation data show that the Amyblood assays are suitable for robust measurement of 1–42 and 1–40 amyloid isoforms in plasma.

The development of the Amyblood assays was motivated by the need for an assay that simultaneously offers high specificity, high sensitivity and high throughput. Therefore, we employed the known specificity of the ADx102, ADx103, and ADx101 antibodies as demonstrated earlier in CSF,^15,16^ together with the sensitivity of the Simoa technology^11^. The promising prototype assay results (eFigure 3) urged us to upscale for widespread validation. Upscaling is challenging since production of larger stock volumes can influence the reagent performance and may affect the sensitivity. Therefore, performance of the prototype and upscaled batch were thoroughly tested. Upscaling did not affect the calibrator curve, reproducibility was demonstrated at Amsterdam UMC and ADx (variation < 20%), and lastly, the assay remained successful in differentiating 20 AD patients and 20 controls (*p* < 0.001). These results indicate that the transformation of our initial prototype to the RTU Amyblood assays was successful.

Our specificity and sensitivity analyses were performed for the Amyblood and Quanterix assays only, since the Amyblood and Euroimmun assays use the same antibodies, but the bead-based Simoa platform can reach higher sensitivity than ELISA. The specificity analyses showed that the Amyblood Abeta_1-42_ assay showed minor cross-reactivity for other proteoforms in sample buffer. At 60 pg/ml Abeta_1-40_ in sample diluent, a non-specific concentration of 2.4 pg/mL was measured with the Amyblood Abeta_1-42_ assay. However, this nonspecific signal could be clinically meaningful, being similar to the group difference of also 2.4 pg/mL Abeta_1-42_ as observed between AD and controls. With the Quanterix Abeta_x-42_ assay, a non-specific signal of 0.9 pg/mL was read at 40 pg/mL Abeta_1-40_, which is three times the group difference of 0.3 pg/mL Abeta_x-42_ as measured with this assay. In addition, a minor increase of 0.8 fold the group difference was measured with the Amyblood Abeta_1-42_ assay when Abeta_3-42_ in sample buffer was incubated, where a large increase of 80 fold the group difference was measured with the Quanterix Abeta_x-42_ assay for this proteoform. The difference in assay specificity could be explained by the Amyblood N-terminal ADx102 (21F2) antibody that is specific for the first amino acid of the Abeta peptide, whereas the Quanterix assay detects N-terminal amino acid 4–10. However, our specificity experiments were performed in sample buffer, whereas a wide variety of endogenous proteins are present in plasma which would make low affinity non-specific binding less likely^17^. Indeed, our selectivity analyses showed a recovery closer to 100% for Amyblood Abeta_1-42_ for higher Abeta_1-42_ concentrations next to the presence of varying concentrations of Abeta_1-40_ peptide. At physiological concentrations of Abeta_1-42_ and Abeta_-40_ in buffer, the %recovery was 125% for Abeta_1-42_. For the Quanterix assay, the Abeta_x-42_ recovery was higher (163%) at physiological concentrations. Our data suggest that the Amyblood Abeta_1-42_ assay had better specificity and selectivity than the Quanterix Abeta_x-42_ assay. Finally, there was no cross reactivity for both Abeta_40_ assays, indicating the high specificity of the 2G3 antibody for Abeta_40_ employed in both formats. Exactly knowing which proteoform is measured as compared to measuring a mixture of full-length and truncated proteoforms might be preferred in clinical trials that target Abeta_1-42_^12^, to be able to specifically map target engagement.

The Amyblood and Quanterix assays could successfully differentiate CSF Abeta_1-42_ positive AD patients and Abeta_1-42_ negative controls. We found that the ELISA ratio could not differentiate AD from controls, but the ELISA Abeta_1-42_ concentration alone could. A recent study comparing the performance of the Abeta_1-42/1–40_ ratio measured with the Amyblood assays and Euroimmun ELISA in non-demented elderly found that both assays could differentiate between amyloid-PET positive and negative participants with similar accuracy^18^. Other studies have also shown that ELISA Abeta_42_ or the Abeta_42/40_ ratio could differentiate between amyloid positive (either by CSF or PET) and negative participants^18–25^. Similar to other studies, we found no correlation between CSF Abeta_1-42_ and plasma Abeta_42_ concentrations, and only a weak correlation with the plasma Abeta_1-42/1–40_ ratio^5,6,20,25^. An explanation could be that amyloid produced peripherally, for example by platelets^26^, distorts the association of Abeta measured in plasma with Abeta produced only by the central nervous system, as measured in CSF. In the CSF, the full length Abeta_1-42_ and Abeta_1-40_ proteoforms and not the n-truncated forms are measured on widely used (automated) platforms to support the specific diagnosis of AD (*e.g.*, Fujirebio Lumipulse, Roche Elecsys). For comparability reasons, it is be preferred to measure this same full-length Abeta_1-42_ and Abeta_1-40_ isoforms in blood.

It has been suggested that SCD cases have a higher risk of converting to AD compared to healthy elderly controls^27^. We carefully selected controls with normal CSF biomarker values, who are not likely to convert^28^. We wish to stress that the focus of our study was to compare analytical performance and clinical samples were included to indicate potential clinical value, since other studies are conducted on early diagnostic use of the Amyblood assays^18,29,30^.

A next step in the development of these assays is to leverage the multiplexing possibilities of Simoa technology and simultaneously detect multiple biomarkers, to reflect different aspects of AD within one assay run, saving time and resources. It is to note, that the results presented in this study, together with the conducted diagnostic Amyblood studies^18,29,30^, resulted in the development of the neurology 4-plex E assay kit by Quanterix, incorporating the Amyblood assays together with the glial fibrillary acidic protein and neurofilament light assays. This availability enables independent and widespread validation. In addition, development of Certified Reference Material is critical to calibrate and compare different amyloid assays and to enable clinical implementation.

## Conclusions

We have developed an exceptionally specific blood test that measures full length Abeta_1-42_ and Abeta_1-40_ using high throughput semi-automated ultrasensitive technology. This study shows that the Amyblood assay has the potential to specifically and sensitively measure the concentrations of full length Abeta_1-42_ and Abeta_1-40_, and as such could be a specific test for target engagement in future clinical trials.

## Methods

### Prototype and Amyblood assay development and transfer

For proof of concept, a prototype assay (supplementary methods) was developed in-house by Amsterdam UMC using a Simoa homebrew assay development kit (Quanterix, MA), utilizing capture antibodies ADx102 (21F12)^14^ for Abeta_1-42_ or ADx103 (2G3)^14^ for Abeta_1-40_ and detector antibody ADx101 (3D6)^14^ for Abeta_1_ (ADx, Ghent, BE). These prototype assays formed the basis for the Amyblood assays that were further developed and upscaled by ADx. The sample diluent formulation and capture antibody conjugation were optimized to improve sensitivity. Reproducibility in %CV was tested on 3 monoclonal antibody lots and on the small batch compared to the upscaled RTU assays (volumes equivalent to 50 assay kits of 96 data points) based on duplicate measurements of the average enzyme per bead (AEB) signal, excluding the blank. Inter- and intra-assay variation were tested in six independent runs by ADx and five runs at Amsterdam UMC based on duplo measurements of two plasma quality control samples. Initial clinical performance of the Abeta_1-42/1–40_ ratio was confirmed in 20 CSF Abeta_1-42_ positive AD cases and 20 CSF Abeta_1-42_ negative controls. Subsequently, the Amyblood RTU kits were shipped to Amsterdam UMC for further assay characterization and clinical validation. The Amyblood reagent preparation and assay set-up are detailed in the Supplementary methods.

### Analytical characterization of the six assays

Analytical validation in all six assays (Abeta_42_ and Abeta_40_ measured with Amyblood, Quanterix, and ELISA) included measurement of the Lower Limit of Quantification (LLOQ) (mean of 16 blanc samples + 10 standard deviations (SD)), linearity upon dilution (15 EDTA plasma samples, three dilutions: dilution factor (df) 4, 6 and 8 for Abeta_1-42_ and df 8, 10, and 12 for Abeta_1-40_), intra-assay %CV (SD of duplicate measurement divided by the mean *100%) of 85 clinical samples and inter-assay %CV of 14 samples over two runs. Additionally, selectivity and specificity were investigated, including physiological, low, medium and high concentrations, detailed in the Supplementary methods.

### Comparison of analytical assay characteristics

To compare the analytical performance of the novel Amyblood assays, we selected two commercially available plasma amyloid immunoassays. The Quanterix Neurology 3-plex A assay kit employs the Simoa HD-1 analyzer, with a different capture antibody for Abeta_x-42_ (H31L21) and the same capture antibody for Abeta_x-40_ (2G3), combined with a different detector antibody (6E10), comprising the RHD motif (aa5-7) that is not N-terminus specific and therefore results in binding of x-42 and x-40^31^. We also selected the Euroimmun ELISA assays that employ the same antibodies as the Amyblood assays to measure Abeta_1-42_ and Abeta_1-40_. The Quanterix and ELISA analytical validation methods are specified in the Supplementary methods.

### Clinical samples

We included 85 participants from the Amsterdam Dementia Cohort^32^ with available EDTA plasma samples in the Biobank, 43 were AD dementia patients and 42 controls (n = 35 with subjective cognitive decline (SCD), n = 7 with psychiatric disease). All subjects visited the Alzheimer Center of the Amsterdam UMC between August 2002 and January 2017 for extensive dementia screening that consisted of neurological, physical, and neuropsychological evaluation, electroencephalography, brain magnetic resonance imaging, and CSF AD biomarker analysis^32^. The diagnosis was made upon multidisciplinary consensus based on applicable clinical criteria^3,33^. CSF Abeta_1-42_, P-tau181, and T-tau were measured using Innotest ELISA (Fuijirebio, Ghent, BE) by research staff blinded for clinical diagnoses^34^. CSF Abeta_1-42_ concentrations were adjusted for the drift in CSF biomarker analyses that occurred over the years and subsequently dichotomized as CSF amyloid positive (≤ 813 pg/ml Abeta_1-42_) and amyloid negative (> 813 pg/ml Abeta_1-42_)^35^. All AD-dementia cases were selected to be CSF amyloid positive, and all controls were required to be CSF amyloid negative (Table 1).

**Table 1:** Cohort characteristics and biomarker values in the clinical validation.

	Cohort	Controls (42)	AD (43)	Total (85)	*p*
	Sex, M/F	25/17	21/22	39/46	0.33
M (SD)	Age, y	61.7 (5)	68.1 (6)	64.9 (6)	< *0.001*
M (SD)	MMSE	27.2 (3)	22.0 (5)	24.6 (5)	< *0.001*
n		42	42	84	
M (IQR)	CSF Tau (pg/mL)	223 (117)	615 (399)	427 (395)	< *0.001*
n		42	43	85	
M (IQR)	CSF P-tau (pg/mL)	39 (15)	89 (37)	63 (36)	< *0.001*
n		41	43	84	
M (IQR)	CSF Abeta 42 (pg/mL)	1132 (308)	658 (153)	804 (475)	< *0.001*
n		42	43	85	

Abeta, Amyloid beta; CSF, Cerebrospinal fluid; ELISA, Enzyme-Linked Immuno Sorbent Assay; MMSE, Mini-Mental State Examination; pTau, phosphorylated tau. M (SD) shows the mean value and standard deviation. M (IQR) shows the median value and interquartile range. P values show the difference between the CSF Aβ negative and the CSF Aβ positive group. Significant p-values are shown in italics (*p* < 0.05).

EDTA-plasma samples were obtained through venipuncture. After 10-min centrifugation at 1800xg within 2 h, plasma was aliquoted in 0.5 mL polypropylene tubes and stored at − 80 °C. Samples were thawed at room temperature and centrifuged at 14,000xg prior to analyses.

Written informed consent to use medical data and biomaterials for research purposes was in place. These and the experimental protocol were in accordance with and approved by the Amsterdam UMC ethical committee, location VUmc, and in accordance with the Helsinki Declaration act of 1975.

### Plasma amyloid measurement

Abeta_1-42_ and Abeta_1-40_ were measured with the Amyblood kit on the Simoa HD-1 analyzer and by Euroimmun ELISA. Concentrations of Abeta_x-42_ and Abeta_x-40_ were measured with the Neurology 3-Plex assay A (Quanterix) on the Simoa HD-1 analyzer. All samples were manually diluted (eTable 1) and analyzed in duplicate, following manufacturer’s instructions.

### Statistical analysis

Differences in Abeta_42_ and Abeta_40_ concentrations between patients with AD and controls were tested with linear regression analysis for group differences, both unadjusted and adjusted for sample storage time, sample run, age, and sex. The Abeta_42/40_ ratios measured with the Amyblood and Quanterix assays were normally distributed. The Abeta_42/40_ ratios measured with the ELISA assay were not normally distributed, neither after natural log transformation, nor after excluding the extreme outlier. Therefore, Spearman correlations were used to investigate all correlations. *P* < 0.05 was considered statistically significant. All analyses were performed using IBM SPSS Statistics, version 26 and graphs were constructed using R version 3.5.3.

### Ethical approval and consent to participate

Written consent to use medical data and biomaterials for research purposes was in place, in accordance with the ethical committee of the Amsterdam UMC, location VUmc, and with the Helsinki Declaration act of 1975.

### Consent for publication

All authors approved this manuscript for publication.

## Supplementary Information

Supplementary Information

## Data Availability

The datasets used and analyzed during the current study can be made available by the corresponding author upon reasonable request.

## Abbreviations

Abeta: Amyloid beta
AD: Alzheimer’s disease
AEB: Average enzyme per bead
AUC: Area under the curve
CSF: Cerebrospinal fluid
LLOQ: Lower Limit of Quantification
MCI: Mild Cognitive Impairment
PET: Positron Emission Tomography
RTU: Ready to use
SCD: Subjective Cognitive Decline
