# Supplementary material for: Highly specific and ultrasensitive plasma test detects Abeta(1–42) and Abeta(1–40) in Alzheimer’s disease
Source: Sci Rep. 2021 May 6;11:9736. doi: 10.1038/s41598-021-89004-x (PMC8102604; doi:10.1038/s41598-021-89004-x)
Supplement: Supplementary file 1 — Supplementary Information [file 41598_2021_89004_MOESM1_ESM.pdf]

# Highly specific and ultrasensitive plasma test detects Abeta(1-42) and Abeta(1-40) in Alzheimer's disease

Elisabeth H. Thijssen, MSc,[1*] Inge M.W. Verberk, MSc,[1*] Jeroen Vanbrabant, PhD,[2] Anne Koelewijn, MSc,[1] Hans Heijst, BSc,[1] Philip Scheltens, MD, PhD,[3] Wiesje van der Flier, PhD,[3] Hugo Vanderstichele, PhD,[4] Erik Stoops, Eng,[2] Charlotte E. Teunissen, PhD[1]

1. Neurochemistry Laboratory, Department of Clinical Chemistry, Amsterdam Neuroscience, Vrije Universiteit Amsterdam, Amsterdam UMC, Amsterdam, The Netherlands

2. ADx NeuroSciences, Ghent, Belgium

3. Alzheimer Center Amsterdam, Department of Neurology, Amsterdam Neuroscience, Vrije Universiteit Amsterdam, Amsterdam UMC, Amsterdam, The Netherlands

4. Biomarkable, Gent, Belgium

* Shared first authorship

## Supplementary Methods

### Prototype assays reagent preparation and assay set-up

For the prototype assays, paramagnetic carboxylated beads (Quanterix) were conjugated with ADx102 (21F12) monoclonal antibody (mAb) for $Abeta_{42}$ and ADx103 (2G3) for $Abeta_{40}$. Biotinylated mAb ADx101 (3D6), specific for detection of $Abeta_{1}$, was provided by ADx NeuroSciences with a Ab/biotin ratio of 8. Both the assay for $Abeta_{1-42}$ and $Abeta_{1-40}$ were performed on the fully automated Quanterix Simoa HD-1 with a 2-step protocol. Samples had dilution factor (df) 4 for $Abeta_{1-42}$ and df10 for $Abeta_{1-40}$, needing respectively 60 µL and 24 µL sample with dead volume per well. The sample dilution buffer was similar to the calibrator diluent and consisted of 1xPBS with 1% milk, 0,1% Tween20 and 333 µg/mL TRU-block (Meridian Life Sciences). The first incubation step of the sample with the beads was 80 cadences for $Abeta_{1-42}$ and 47 cadences (1 cadence = 45 s) for $Abeta_{1-40}$. After washing, the second incubation step with 100 µL 50 pM streptavidin-β-galactosidase (Quanterix) was 7 cadences for both assays. Prior to reading Resorufin-β-D-galactopyranoside (Quanterix) was added.

### Amyblood assays reagent preparation and assay set-up

Reagents of the upscaled batch were prepared as follows. For capture bead conjugation, 2.56*10^9 beads in 2 mL conjugation volume were activated using 0.1 mg/mL 1-Ethyl-3-(3-Dimethylaminopropyl)carbodiimide (EDAC) for 20 minutes at 4 °C and conjugated for 2 hours at RT with 0.2 mg/mL ADx102 (21F12) or ADx103 (2G3). Beads were blocked after couping using Quanterix Blocking buffer for 45 minutes at RT. Detector antibody ADx101 (3D6) was biotinylated using sNHS-Lc-Bio (Thermo Fisher) in a 8x molar excess at 1 mg/mL antibody concentration in 50 mM boric acid/sodium tetraborate buffer pH 8.5 and the biotinylation reaction was quenched by addition of 20 mM Tris. Capture beads and detector antibody were subsequently diluted in sample diluent (1xPBS w. 0.1% Casein, 0.1% Tween-20 and 50 µg/mL HBR-1 heterophylic blocker (Scantibodies, USA)) as RTU solutions. Bead RTU solution contained 2*10^7 beads per analyte from which 50% were helper beads (Quanterix). Detector RTU solution contained 0.1 µg/mL biotinylated ADx101 (3D6) in sample diluent. Calibrator RTU solutions were prepared using a 7-point 1:2 serial dilution series of 64 pg/mL $Abeta_{1-42}$ or $Abeta_{1-40}$ peptide (custom made by Polypeptide, BE) in sample diluent.

The Amyblood assays are 2-step assays combining 25 µL RTU beads, 20 µL RTU Detector and 100 µL RTU calibrator or pre-diluted EDTA plasma in sample diluent (dilution factor (df) 4 for $Abeta_{1-42}$; df10 for $Abeta_{1-40}$) in a first incubation step of 160 cadences (2 hours) on the Simoa HD-1 analyzer. After washing, a second incubation step of 7 cadences (5.25 minutes) followed wherein the 100 µL 50 pM enzyme conjugate streptavidin-β-galactosidase (Quanterix) labels the immunocomplex, followed by addition of the substrate resorufin β-D-galactopyranoside (RGP) for the fluorescent read-out. Research staff was blinded for clinical diagnoses.

**Specificity and selectivity analyses**

Specificity is the ability of an assay to distinguish between the analyte that the assay is intended to measure, and structurally similar analytes. We tested specificity of the Amyblood $Abeta_{1-42}$ assay by measuring the assay signal in response to increasing concentrations of 6 pg/mL, 25 pg/mL and 60 pg/mL of the fragments $Abeta_{1-40}$, $Abeta_{1-43}$, $Abeta_{2-42}$ and $Abeta_{3-42}$ (custom made by Polypeptide, BE) in sample buffer. We tested specificity of the Amyblood $Abeta_{1-40}$ assay by measuring the assay signal in response to increasing concentrations of 6 pg/mL, 25 pg/mL and 60 pg/mL of the fragments $Abeta_{1-42}$, $Abeta_{1-38}$, $Abeta_{1-39}$ and $Abeta_{11-40}$ (custom made by Polypeptide, BE) in sample buffer. The AEBs

from Abeta1-42 and Abeta1-40 in buffer measured with the Amyblood assays were extrapolated from the calibration curve to calculate the corresponding concentrations. The AEBs from $Abeta_{1-42}$ and $Abeta_{1-40}$ in buffer measured with the Quanterix triplex were calculated derived from the linear measurements in 4, 25, and 130 pg/mL for $Abeta_{1-42}$ and 1, 15, and 40 pg/mL for $Abeta_{1-40}$. These AEBs were then extrapolated from the calibration curve to calculated the corresponding concentrations.

Selectivity is the ability of an assay to accurately detect an analyte concentration, in the presence of another, independent analyte that is expected to be present in a test sample. We tested selectivity by measuring the signal of one analyte in sample diluent (either $Abeta_{1-42}$ or $Abeta_{1-40}$), in the presence of a known concentration of the other analyte. The added concentrations were selected to include the physiological concentrations present in plasma, as well as concentrations lower and higher than the physiological concentrations. For $Abeta_{1-42}$ selectivity, we spiked either 6 pg/mL, 25 pg/mL or 60 pg/mL $Abeta_{1-42}$ in sample diluent, combined with 0 pg/mL (reference condition), 3 pg/mL, 15 pg/mL and 60 pg/mL $Abeta_{1-40}$. For $Abeta_{1-40}$ selectivity, we spiked either 3 pg/mL, 15 pg/mL or 60 pg/mL $Abeta_{1-40}$ in sample diluent, combined with 0 pg/mL (reference condition), 6 pg/mL, 25 pg/mL or 60 pg/mL $Abeta_{1-42}$.

**Quanterix and ELISA assay validation**

The Quanterix commercial assays were validated at Amsterdam UMC. Linearity was based on three native EDTA plasma samples, six dilutions were assessed that ranged from df4 to df12500. Intra-assay %CV, LLOQs, specificity and sensitivity were assessed with the same method as the Amyblood assays. Inter-assay %CV was based on 3 neat plasma QC samples: QC Low and QC Medium were measured in 4 different runs on different days. QC high was measured in 3 different runs on different days. We tested specificity of the Quanterix $Abeta_{x-42}$ assay by measuring the assay signal in response to increasing concentrations of 1 pg/mL, 15 pg/mL and 40 pg/mL of the fragments $Abeta_{1-40}$, $Abeta_{1-43}$, $Abeta_{2-42}$, and $Abeta_{3-42}$ in sample buffer. We tested specificity of the Quanterix $Abeta_{x-40}$ assay by measuring the assay signal in response to increasing concentrations of 4 pg/mL, 25 pg/mL and 130 pg/mL of the fragments $Abeta_{1-42}$, $Abeta_{1-38}$, $Abeta_{1-39}$, and $Abeta_{11-40}$ in sample buffer. For Quanterix $Abeta_{x-42}$ selectivity, we spiked either 1 pg/mL, 15 pg/mL or 40 pg/mL $Abeta_{1-42}$ in sample diluent, combined with 0 pg/mL (reference condition), 4 pg/mL, 25 pg/mL and 130 pg/mL $Abeta_{1-40}$. For $Abeta_{x-}$

$_{40}$ selectivity, we spiked either 4 pg/mL, 25 pg/mL or 130 pg/mL Abeta$_{1-40}$ in sample diluent, combined with 0 pg/mL (reference condition), 1 pg/mL, 15 pg/mL or 40 pg/mL Abeta$_{1-42}$.

The ELISA LLOQ's were based on the limit of blank data provided by Euroimmun and calculated as the average concentration of 24 blank measurements + 10 standard deviations. The linearity of the ELISA assays was based on data provided by Euroimmun on two EDTA plasma pools spiked with recombinant Abeta$_{1-42}$ or Abeta$_{1-40}$ at different concentrations and one native EDTA plasma sample. Ten dilutions were assessed, from df 1 to df 10. We expect the ELISA assay to perform similar to the Amyblood, since both assays use the same antibodies, and we therefore did not include that assay in the specificity and selectivity studies.

**Supplementary results**

### Prototype and Amyblood assay development and transfer

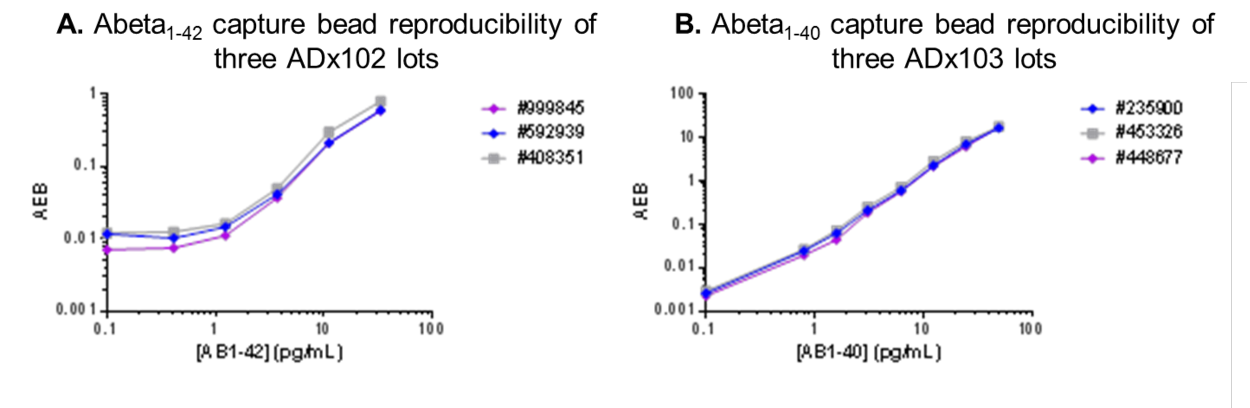


**eFigure 1. Lot to lot variation tested on calibrator curves of three different capture antibody lots.** **A.** The mean variation in AEB values between the three different antibody lots tested for Abeta$_{1-42}$ was 19%. **B.** The mean variation in AEB values between the three different antibody lots tested for Abeta$_{1-40}$ was 14%.

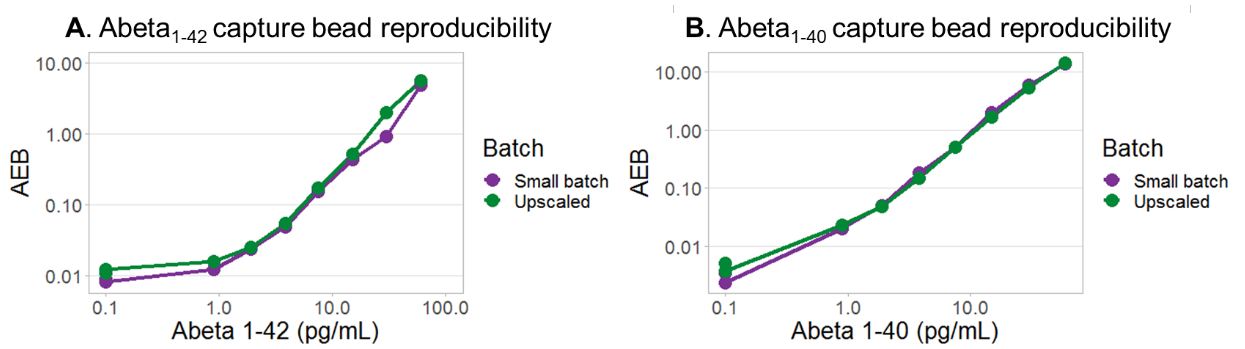


**eFigure 2. Calibrator curve comparison between small and upscaled bead batch**
**A.** Mean variation in AEB values between the small batch and upscaled batch is 15.9% for Abeta$_{1-42}$. **B.** Mean variation in AEB values between the small batch and upscaled batch is 7.0% for Abeta$_{1-40}$. Upscaling to bead conjugation volumes from 100µL to 2 mL did not affect calibrator AEB curves.

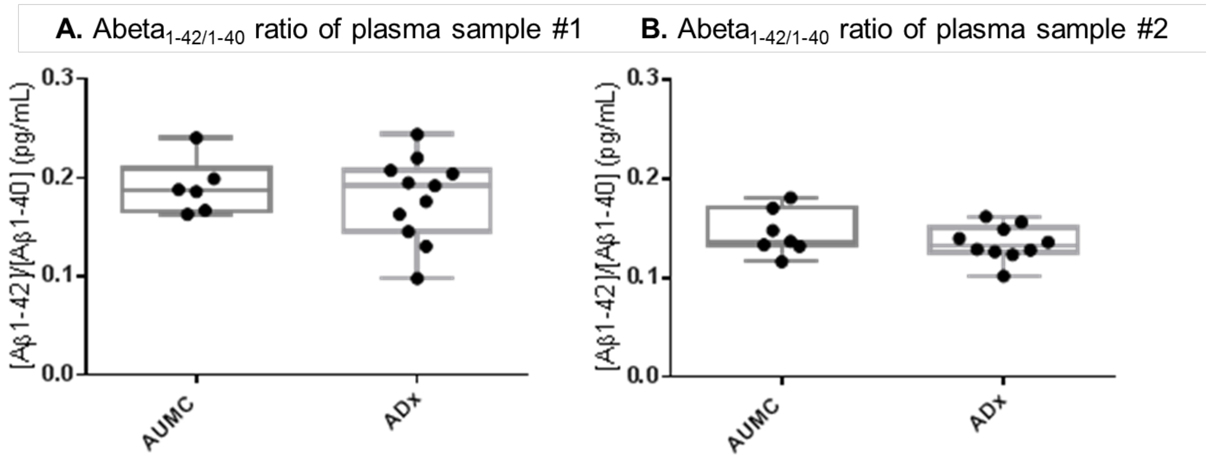


**eFigure 3. Two neat plasma samples measured with the Amyblood assays at AUMC and ADx.**
**A.** The mean Abeta$_{1-42/1-40}$ ratio of plasma sample 1 was 0.17 ADx and 0.19 at AUMC. **B.** The mean Abeta$_{1-42/40}$ ratio of plasma sample 2 was 0.13 ADx and 0.14 at Amsterdam UMC. Mean intra-assay %CV of the Abeta$_{1-42/40}$ ratio of two neat samples was 3.5% and 2.0% at ADx and 3.9% and 2.2% at Amsterdam UMC. Mean inter-assay %CV of the Abeta$_{1-42/40}$ ratio was 23% and 13% at ADx and 14% for both samples at Amsterdam UMC. Overall mean inter-assay and inter-center variation of the Abeta ratio was 17.2%, combining all results. Abeta: amyloid beta; AUMC: Amsterdam University Medical Centers; ADx; ADx Neurosciences

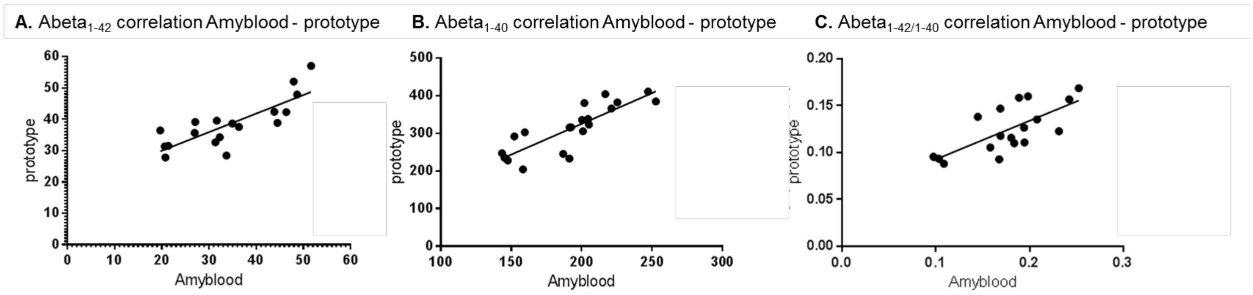


**eFigure 4. Correlations between amyloid concentrations measured with the prototype and Amyblood**
**A.** Correlation between the prototype and Amyblood measurements of Abeta$_{1-42}$ (R=0.77 (95%CI:0.46-0.91), $p$<0.001). **B**. Correlation between the prototype and Amyblood measurements of Abeta$_{1-40}$ (R=0.89 (95% CI: 0.74-0.96), $p$<0.001). **C**. Correlation between the prototype and Amyblood measurements of the Abeta$_{1-42/1-40}$ ratio (R=0.69 (95% CI: 0.32-0.88), $p$=0.0014). Abeta: amyloid beta.

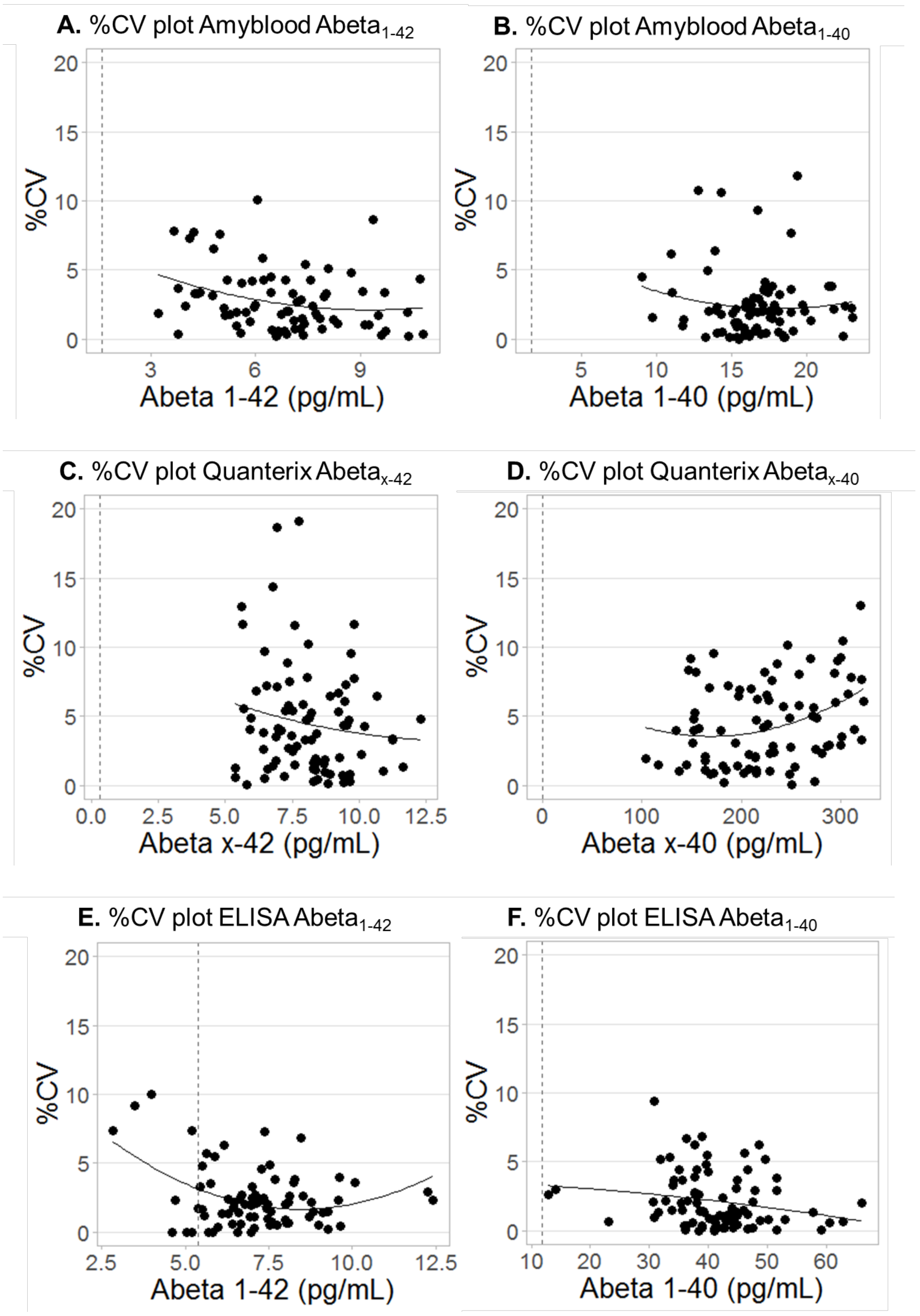


**eFigure 5. Percentage coefficient of variation in patient samples measured with three immunoassays. A**. Abeta$_{1-42}$ measured with the Amyblood assay. For visualization a sample with %CV of 26.1 and Abeta$_{-42}$ of 5.2 pg/mL (below LLOQ) was excluded. This concentration was below LLOQ. **B**. Abeta$_{1-40}$ measured with the Amyblood assay. **C**. Abeta$_{x-42}$ measured with the Quanterix assay. **D**. Abeta$_{1-40}$ measured with the Quanterix assay. **E**. Abeta$_{1-42}$ measured with the ELISA assay. Eight samples were measured below LLOQ. **F**. Abeta$_{1-40}$ measured with the ELISA assay. Dashed line represents the LLOQ per assay

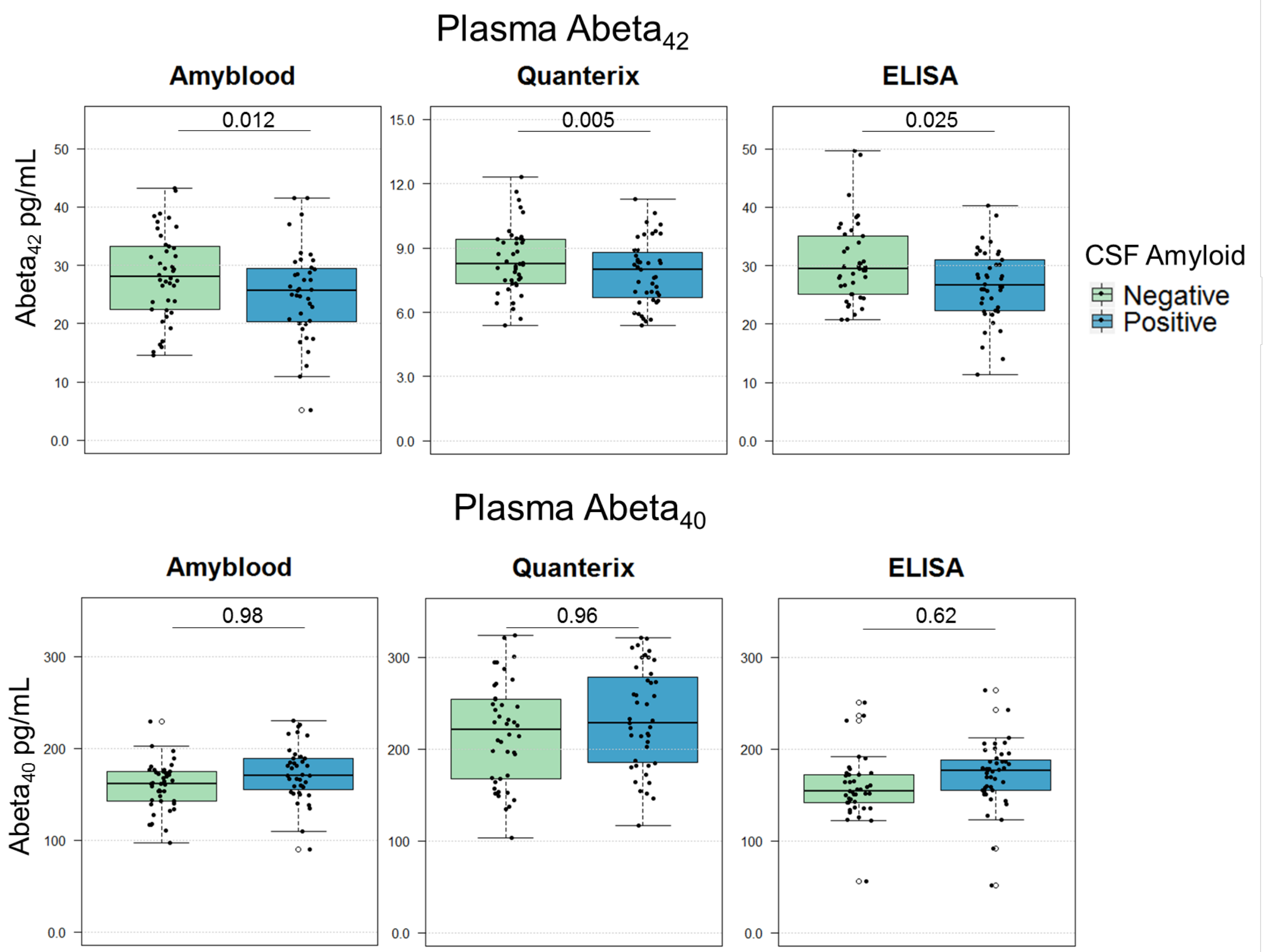


**eFigure 6. Plasma Abeta$_{42}$ and Abeta$_{40}$ measured with three different immunoassays**
Plasma Abeta$_{42}$ and Abeta$_{40}$ measured in CSF Abeta$_{42}$ negative and CSF Abeta$_{42}$ positive groups, using the Amyblood and Quanterix Simoa assays and the Euroimmun ELISA. P-values are corrected for age, sex, sample batch, and sample storage time.

| | Abeta 42 | | | Abeta 40 | | |
|---|---|---|---|---|---|---|
| | Amyblood | Quanterix | ELISA | Amyblood | Quanterix | ELISA |
| Sample dilution | 4x manually | 4x manually | 4x manually | 10x manually | 4x manually | 4x manually |
| Sample volume | 60 µL | 86 (in total) | 60 uL | 24 µL | 86 uL (in total) | 60 uL |
| Capture antibody | ADx102 (21F12) | H31L21 | ADx102 (21F12) | ADx103 (2G3) | 2G3 | ADx103 (2G3) |
| Detector antibody | ADx101 (3D6) | 6E10 | ADx101 (3D6) | ADx101 (3D6) | 6E10 | ADx101 (3D6) |
| Calibration curve | 1-64 pg/mL | 0-60 pg/mL | 1-75 pg/mL | 1-64 pg/mL | 0-140 pg/mL | 1-40 pg/mL |
| LLOQ | 1.6 pg/mL | 0.34 pg/mL | 5.4 pg/mL | 1.7 pg/mL | 0.16 pg/mL | 11.9 pg/mL |
| Linearity | 104% | 122% | 100% | 96% | 103% | 100% |
| %intra-assay CV | 3.1% | 2.0% | 2.7% | 2.4% | 1.5% | 2.1% |
| #samples <LLOQ | 1 | 0 | 8 | 0 | 0 | 0 |

**eTable 1: Technical specifications of the different assay versions**
The Amyblood and Quanterix assays were performed using the Quanterix Simoa. The sample volume is for duplo measurement including dead volume. Abeta; amyloid beta, LLOQ; Lower limit of quantification.

| Amyblood assay | Spiked analyte | Concentration | Conc. Abeta 1-42 | Stdev |
|---|---|---|---|---|
| Amyblood Abeta 1-42 | Abeta 1-42 | 6 pg/mL | 6.0 | NA |
| Amyblood Abeta 1-42 | Abeta 1-42 | 25 pg/mL | 25.1 | NA |
| Amyblood Abeta 1-42 | Abeta 1-42 | 60 pg/mL | 59.8 | NA |
| Amyblood Abeta 1-42 | Abeta 1-40 | 6 pg/mL | 1.7 | 0.04 |
| Amyblood Abeta 1-42 | Abeta 1-40 | 25 pg/mL | 1.8 | 0.11 |
| Amyblood Abeta 1-42 | Abeta 1-40 | 60 pg/mL | 2.4 | 0.07 |
| Amyblood Abeta 1-42 | Abeta 1-43 | 6 pg/mL | 1.8 | 0.26 |
| Amyblood Abeta 1-42 | Abeta 1-43 | 25 pg/mL | 1.8 | 0.15 |
| Amyblood Abeta 1-42 | Abeta 1-43 | 60 pg/mL | 2.1 | 0.25 |
| Amyblood Abeta 1-42 | Abeta 2-42 | 6 pg/mL | 1.8 | 0.18 |
| Amyblood Abeta 1-42 | Abeta 2-42 | 25 pg/mL | 1.8 | 0.18 |
| Amyblood Abeta 1-42 | Abeta 2-42 | 60 pg/mL | 2.1 | 0.09 |
| Amyblood Abeta 1-42 | Abeta 3-42 | 6 pg/mL | 2.0 | 0.18 |
| Amyblood Abeta 1-42 | Abeta 3-42 | 25 pg/mL | 1.8 | 0.16 |
| Amyblood Abeta 1-42 | Abeta 3-42 | 60 pg/mL | 1.8 | 0.00 |

| Amyblood assay | Spiked analyte | Concentration | Conc. Abeta 1-40 | Stdev |
|---|---|---|---|---|
| Amyblood Abeta 1-40 | Abeta 1-40 | 6 pg/mL | 6.0 | NA |
| Amyblood Abeta 1-40 | Abeta 1-40 | 25 pg/mL | 25.0 | NA |
| Amyblood Abeta 1-40 | Abeta 1-40 | 60 pg/mL | 60.0 | NA |
| Amyblood Abeta 1-40 | Abeta 1-42 | 6 pg/mL | NA | NA |
| Amyblood Abeta 1-40 | Abeta 1-42 | 25 pg/mL | NA | NA |
| Amyblood Abeta 1-40 | Abeta 1-42 | 60 pg/mL | NA | NA |
| Amyblood Abeta 1-40 | Abeta 1-38 | 6 pg/mL | NA | NA |
| Amyblood Abeta 1-40 | Abeta 1-38 | 25 pg/mL | NA | NA |
| Amyblood Abeta 1-40 | Abeta 1-38 | 60 pg/mL | NA | NA |
| Amyblood Abeta 1-40 | Abeta 1-39 | 6 pg/mL | NA | NA |
| Amyblood Abeta 1-40 | Abeta 1-39 | 25 pg/mL | NA | NA |
| Amyblood Abeta 1-40 | Abeta 1-39 | 60 pg/mL | NA | NA |
| Amyblood Abeta 1-40 | Abeta 11-40 | 6 pg/mL | NA | NA |
| Amyblood Abeta 1-40 | Abeta 11-40 | 25 pg/mL | NA | NA |
| Amyblood Abeta 1-40 | Abeta 11-40 | 60 pg/mL | NA | NA |

| Quanterix assay | Spiked analyte | Concentration | Conc. Abeta x-42 | Stdev |
|---|---|---|---|---|
| Quanterix Abeta x-42 | Abeta 1-42 | 1 pg/mL | NA | NA |
| Quanterix Abeta x-42 | Abeta 1-42 | 15 pg/mL | 2.9 | NA |
| Quanterix Abeta x-42 | Abeta 1-42 | 40 pg/mL | 8.4 | NA |
| Quanterix Abeta x-42 | Abeta 1-40 | 1 pg/mL | 0.2 | 0.11 |
| Quanterix Abeta x-42 | Abeta 1-40 | 15 pg/mL | 0.9 | 0.23 |
| Quanterix Abeta x-42 | Abeta 1-40 | 40 pg/mL | 0.9 | 0.01 |
| Quanterix Abeta x-42 | Abeta 1-43 | 1 pg/mL | 0.4 | 0.20 |
| Quanterix Abeta x-42 | Abeta 1-43 | 15 pg/mL | 0.2 | 0.01 |
| Quanterix Abeta x-42 | Abeta 1-43 | 40 pg/mL | 0.5 | 0.20 |
| Quanterix Abeta x-42 | Abeta 2-42 | 1 pg/mL | 0.2 | 0.11 |
| Quanterix Abeta x-42 | Abeta 2-42 | 15 pg/mL | 1.9 | 0.09 |
| Quanterix Abeta x-42 | Abeta 2-42 | 40 pg/mL | 4.4 | 0.07 |
| Quanterix Abeta x-42 | Abeta 3-42 | 1 pg/mL | 0.6 | 0.01 |
| Quanterix Abeta x-42 | Abeta 3-42 | 15 pg/mL | 7.6 | 0.19 |
| Quanterix Abeta x-42 | Abeta 3-42 | 40 pg/mL | 24.7 | 0.26 |

| Quanterix assay | Spiked analyte | Concentration | Conc. Abeta x-40 | Stdev |
|---|---|---|---|---|
| Quanterix Abeta x-40 | Abeta 1-40 | 6 pg/mL | 0.1 | NA |
| Quanterix Abeta x-40 | Abeta 1-40 | 25 pg/mL | 2.0 | NA |
| Quanterix Abeta x-40 | Abeta 1-40 | 60 pg/mL | 63.4 | NA |
| Quanterix Abeta x-40 | Abeta 1-42 | 6 pg/mL | NA | NA |
| Quanterix Abeta x-40 | Abeta 1-42 | 25 pg/mL | NA | NA |
| Quanterix Abeta x-40 | Abeta 1-42 | 60 pg/mL | NA | NA |
| Quanterix Abeta x-40 | Abeta 1-38 | 6 pg/mL | NA | NA |
| Quanterix Abeta x-40 | Abeta 1-38 | 25 pg/mL | NA | NA |
| Quanterix Abeta x-40 | Abeta 1-38 | 60 pg/mL | NA | NA |
| Quanterix Abeta x-40 | Abeta 1-39 | 6 pg/mL | NA | NA |
| Quanterix Abeta x-40 | Abeta 1-39 | 25 pg/mL | NA | NA |
| Quanterix Abeta x-40 | Abeta 1-39 | 60 pg/mL | NA | NA |
| Quanterix Abeta x-40 | Abeta 11-40 | 6 pg/mL | NA | NA |
| Quanterix Abeta x-40 | Abeta 11-40 | 25 pg/mL | NA | NA |
| Quanterix Abeta x-40 | Abeta 11-40 | 60 pg/mL | NA | NA |

**eTable 2. Specificity results Amyblood and Quanterix assays**

The concentrations of Abeta$_{1-42}$ (pg/mL) measured with the Amyblood assay and the concentrations Abeta$_{x-42}$ (pg/mL) measured with the Quanterix assay. The signal obtained with the Amyblood Abeta$_{1-40}$ assay and Quanterix Abeta$_{x-40}$ assay was too low to detect and therefore not included in this table.
Abeta: Amyloid beta

| Amyblood assay | Added Abeta 1-40 | Added Abeta 1-42 | %recovery Abeta 1-42 | Stdev |
|---|---|---|---|---|
| Amyblood Abeta 1-42 | 0 pg/mL | 6 pg/mL | 100 | 0.0 |
| Amyblood Abeta 1-42 | 3 pg/mL | 6 pg/mL | 115 | 20.6 |
| Amyblood Abeta 1-42 | 15 pg/mL | 6 pg/mL | 125 | 2.9 |
| Amyblood Abeta 1-42 | 60 pg/mL | 6 pg/mL | 154 | 3.0 |
| Amyblood Abeta 1-42 | 0 pg/mL | 25 pg/mL | 100 | 0.0 |
| Amyblood Abeta 1-42 | 3 pg/mL | 25 pg/mL | 89 | 1.4 |
| Amyblood Abeta 1-42 | 15 pg/mL | 25 pg/mL | 91 | 6.4 |
| Amyblood Abeta 1-42 | 60 pg/mL | 25 pg/mL | 105 | 1.2 |
| Amyblood Abeta 1-42 | 0 pg/mL | 60 pg/mL | 100 | 0.0 |
| Amyblood Abeta 1-42 | 3 pg/mL | 60 pg/mL | 101 | 4.9 |
| Amyblood Abeta 1-42 | 15 pg/mL | 60 pg/mL | 100 | 0.5 |
| Amyblood Abeta 1-42 | 60 pg/mL | 60 pg/mL | 105 | 7.6 |

| Amyblood assay | Added Abeta 1-42 | Added Abeta 1-40 | %recovery Abeta 1-40 | Stdev |
|---|---|---|---|---|
| Amyblood Abeta 1-40 | 0 pg/mL | 3 pg/mL | 100 | 0.00 |
| Amyblood Abeta 1-40 | 6 pg/mL | 3 pg/mL | 113 | 12.82 |
| Amyblood Abeta 1-40 | 25 pg/mL | 3 pg/mL | 109 | 0.82 |
| Amyblood Abeta 1-40 | 60 pg/mL | 3 pg/mL | 110 | 1.52 |
| Amyblood Abeta 1-40 | 0 pg/mL | 15 pg/mL | 100 | 0.00 |
| Amyblood Abeta 1-40 | 6 pg/mL | 15 pg/mL | 110 | 0.13 |
| Amyblood Abeta 1-40 | 25 pg/mL | 15 pg/mL | 94 | 2.43 |
| Amyblood Abeta 1-40 | 60 pg/mL | 15 pg/mL | 95 | 0.18 |
| Amyblood Abeta 1-40 | 0 pg/mL | 60 pg/mL | 100 | 0.00 |
| Amyblood Abeta 1-40 | 6 pg/mL | 60 pg/mL | 102 | 3.22 |
| Amyblood Abeta 1-40 | 25 pg/mL | 60 pg/mL | 103 | 3.68 |
| Amyblood Abeta 1-40 | 60 pg/mL | 60 pg/mL | 96 | 2.72 |

| Quanterix assay | Added Abeta 1-40 | Added Abeta 1-42 | %recovery Abeta x-42 | Stdev |
|---|---|---|---|---|
| Quanterix Abeta x-42 | 0 pg/mL | 1 pg/mL | 100 | 0.00 |
| Quanterix Abeta x-42 | 4 pg/mL | 1 pg/mL | 105 | 2.82 |
| Quanterix Abeta x-42 | 25 pg/mL | 1 pg/mL | 163 | 14.53 |
| Quanterix Abeta x-42 | 130 pg/mL | 1 pg/mL | 1021 | 49.04 |
| Quanterix Abeta x-42 | 0 pg/mL | 15 pg/mL | 100 | 0.00 |
| Quanterix Abeta x-42 | 4 pg/mL | 15 pg/mL | 96 | 11.22 |
| Quanterix Abeta x-42 | 25 pg/mL | 15 pg/mL | 112 | 4.51 |
| Quanterix Abeta x-42 | 130 pg/mL | 15 pg/mL | 188 | 14.20 |
| Quanterix Abeta x-42 | 0 pg/mL | 40 pg/mL | 100 | 0.00 |
| Quanterix Abeta x-42 | 4 pg/mL | 40 pg/mL | 88 | 2.87 |
| Quanterix Abeta x-42 | 25 pg/mL | 40 pg/mL | 106 | 1.55 |
| Quanterix Abeta x-42 | 130 pg/mL | 40 pg/mL | 140 | 14.39 |

| Quanterix assay | Added Abeta 1-42 | Added Abeta 1-40 | %recovery Abeta x-40 | Stdev |
|---|---|---|---|---|
| Quanterix Abeta x-40 | 0 pg/mL | 4 pg/mL | 100 | 0.00 |
| Quanterix Abeta x-40 | 1 pg/mL | 4 pg/mL | 89 | 11.15 |
| Quanterix Abeta x-40 | 15 pg/mL | 4 pg/mL | 86 | 2.39 |
| Quanterix Abeta x-40 | 40 pg/mL | 4 pg/mL | 92 | 10.35 |
| Quanterix Abeta x-40 | 0 pg/mL | 25 pg/mL | 100 | 0.00 |
| Quanterix Abeta x-40 | 1 pg/mL | 25 pg/mL | 94 | 0.18 |
| Quanterix Abeta x-40 | 15 pg/mL | 25 pg/mL | 96 | 13.07 |
| Quanterix Abeta x-40 | 40 pg/mL | 25 pg/mL | 97 | 3.01 |
| Quanterix Abeta x-40 | 0 pg/mL | 130 pg/mL | 100 | 0.00 |
| Quanterix Abeta x-40 | 1 pg/mL | 130 pg/mL | 112 | 0.00 |
| Quanterix Abeta x-40 | 15 pg/mL | 130 pg/mL | 115 | 6.86 |
| Quanterix Abeta x-40 | 40 pg/mL | 130 pg/mL | 106 | 0.35 |

**eTable 3. Selectivity results Amyblood and Quanterix assays**
The percentage recovery (%recovery) of $Abeta_{1-42}$ and $Abeta_{1-40}$ measured with the Amyblood assays, and the %recovery of $Abeta_{x-42}$ and $Abeta_{x-40}$ measured with the Quanterix assays. Abeta: Amyloid beta.

| | Cohort | Controls (42) | AD (43) | Total (85) | *p* |
|---|---|---|---|---|---|
| | *Amyblood* | | | | |
| M (IQR) | Abeta 1-42 (pg/mL) | 28.1 (11) | 25.7 (9) | 27.2 (10) | ***0.012*** |
| n | | 42 | 39 | 81 | |
| M (IQR) | Abeta 1-40 (pg/mL) | 161 (33) | 170.5 (30) | 166.1 (32) | *0.98* |
| n | | 42 | 39 | 81 | |
| M (IQR) | Abeta 1-42/1-40 ratio | 0.17 (0.07) | 0.14 (0.04) | 0.16 (0.05) | ***<0.001*** |
| n | | 42 | 39 | 81 | |
| | *Quanterix* | | | | |
| M (IQR) | Abeta x-42 (pg/mL) | 8.3 (2) | 8.0 (2) | 8.1 (2) | ***0.005*** |
| n | | 42 | 43 | 85 | |
| M (IQR) | Abeta x-40 (pg/mL) | 220.9 (88) | 228.1 (97) | 226.1 (91) | *0.96* |
| n | | 42 | 43 | 85 | |
| M (IQR) | Abeta x-42/x-40 ratio | 0.04 (0.01) | 0.03 (0.01) | 0.04 (0.01) | ***<0.001*** |
| n | | 42 | 43 | 85 | |
| | *ELISA* | | | | |
| M (IQR) | Abeta 1-42 (pg/mL) | 29.4 (10) | 26.6 (9) | 28.2 (8) | ***0.025*** |
| n | | 41 | 42 | 83 | |
| M (IQR) | Abeta 1-40 (pg/mL) | 154.2 (30) | 177.0 (35) | 164.0 (36) | 0.62 |
| n | | 42 | 43 | 85 | |
| M (IQR) | Abeta 1-42/1-40 ratio | 0.19 (0.04) | 0.16 (0.05) | 0.17 (0.04) | *0.24* |
| n | | 41 | 42 | 83 | |

**eTable 4: Cohort characteristics and biomarker values in the clinical validation**
Abeta, Amyloid beta; ELISA, Enzyme-Linked Immuno Sorbent Assay; M (IQR) shows the median value and interquartile range. P values show the difference between the CSF Aβ negative and the CSF Aβ positive group. Plasma biomarker p-values are adjusted for sample storage time, sample run, age, and sex. Significant p-values are shown in bold ($p$<0.05).
